# Self-Efficacy in Nursing Competencies during Students’ Clinical Practicum: The Development of a Self-Assessment Scale

**DOI:** 10.3390/nursrep14030173

**Published:** 2024-09-10

**Authors:** Juan Arribas-Marín, Calixto Plumed-Moreno, Vicente Hernández-Franco

**Affiliations:** 1 San Juan de Dios University School of Nursing and Physical Therapy, Comillas Pontifical University, 28350 Madrid, Spain; cplumed@comillas.edu; 2 San Juan de Dios Foundation, 28036 Madrid, Spain; 3 Faculty of Humanities and Social Sciences, Comillas Pontifical University, 28049 Madrid, Spain; vhernandez@comillas.edu

**Keywords:** nursing education, clinical competence, clinical practice, latent class analysis, questionnaire design, validation study

## Abstract

The evaluation of the competencies corresponding to the different professional profiles of future nursing graduates is fundamental to their training. In this regard, students’ self-evaluation must be part of their training. This study aimed to develop and psychometrically test the Perceived Self-Efficacy in Nursing Competencies (PSENC) Scale. This study was conducted in two phases: selecting and adjusting items and assessing the instrument’s psychometric properties. A sample of 1416 students completed the scale online. Exploratory factor and confirmatory factor analyses were conducted. Inferential analysis was carried out. The exploratory factor analysis of the PSENC scale with 20 items resulted in five factors (76.3% of variance). All factors showed Cronbach’s alpha coefficients > 0.70. The confirmatory factor analysis measurement model showed satisfactory and adequate goodness-of-fit indices. The developed scale showed the psychometric adequacy and usefulness to the self-assessment of nursing students regarding their self-efficacy expectations in competencies during their clinical practicum. This study was not registered.

## 1. Introduction

Bandura developed the self-efficacy theory in the framework of the social cognitive theory, considering efficacy beliefs as the basis of human behaviors. The author defined perceived self-efficacy as “people’s judgments of their capabilities to organize and execute courses of action required to attain designated types of performances” [1] (p. 391). Bandura states that people’s beliefs about their abilities are not manifested uniformly as if they were a general characteristic [2]. On the contrary, the effectiveness varies with different domains of functioning and even with other aspects of the activity of a specific domain. Therefore, it suggests that the construction and validation of a self-efficacy scale require strong theoretical support regarding the domain of functioning it intends to evaluate. In this study, self-efficacy theory is the framework for investigating students’ perception of self-efficacy in nursing competencies in the context of the clinical nursing practicum.

Competencies have been configured as crucial elements in evaluating nursing students’ professional development. Consequently, many research studies have focused on constructing tools for assessing the competence level of professionals and students. In this respect, Meretoja and Leino-Kilpi reviewed the literature related to instruments for assessing the competence of practicing nurses [3]. This study was the basis for the subsequent development of the Nurse Competence Scale [4], one of the most used generic scales to assess registered nurses’ competence. During that period, the European Higher Education Area (EHEA) was built, where competencies were configured as the basis for the different professional profiles of future university graduates. The Tuning Project, developed during the EHEA configuration process, promoted international working groups in several academic disciplines. The Tuning Project “acknowledges to the full the importance of building-up a developing subject specific knowledge and skills as the basis for university degree programmes…” [5] (p. 32).

These working groups assumed “Competences represent a dynamic combination of knowledge, understanding, skills, abilities and values. Fostering these competences is the object of educational programmes. Competences will be formed in various course units and assessed at different stages. Competences are obtained by the student.” [5] (p. 14). In the Tuning Project framework, the nursing area’s working group proposed 40 specific competencies for the nursing degree. For the students’ progressive acquisition of this set of competencies, learning in professional settings has been configured as the core and integrating element of training projects.

Taking 40 specific competencies initially defined by the Tuning Project for the nursing degree as a reference, Blažun et al. [6] surveyed the perception of competence in postgraduates studying a master’s degree at the University of Maribor, Slovenia. In their conclusions, the authors raised the need to reinforce aspects related to interdisciplinary communication, teamwork, and collaborative work in future curricular designs.

In the framework of evaluating the development of nursing competencies in the clinical practicum, Helminen et al. [7] conducted a literature review on studies that, in previous years, addressed the summative or final assessment process of student nurses’ clinical practice. From the 23 articles selected for the review, they considered that the evaluation processes presented a lack of consistency. Similarly, Reljic et al. [8] performed a literature review on clinical nursing skills assessment and competence methods. From 12 studies developed in clinical settings and clinical skills laboratories, the authors concluded that there “is a need for further research to develop a holistic clinical assessment tool with a reasonable level of validity and reliability” [8] (p. 63).

Several recent studies have focused on developing skill assessment tools for nurses. Nilsson et al. [9] developed the Nurse Professional Competence (NPC) Scale on a sample of recently graduated Swedish nursing students. Item selection was based on national and international professional competence requirements for nurses. The scale is made up of 88 items that were grouped into eight factors. Subsequently, they validated the NPC Scale Short Form (NPC Scale-SF), a short version composed of 35 items grouped into six factors [10].

Based on the Nordic Advanced Practice Nursing Model, Finnbakk et al. [11] developed the Professional Nurse Self-Assessment Scale (ProffNurse SAS) aiming at facilitating the self-assessment of nurses’ clinical competence. The study was conducted on a sample of 357 registered nurses who worked the Norwegian health system’s long-term and home-care contexts. The scale comprises 51 items grouped into six components: direct clinical practice, professional development, ethical decision-making, clinical leadership, cooperation and consultation, and critical thinking.

In a study involving undergraduate nursing seniors (N = 252) from four Canadian universities, Kennedy et al. [12] developed the Nursing Competence Self-Efficacy Scale (NCSES). Based on the construction of the scale in Entry-Level Competencies for Registered Nurses of Canada, the definitive scale comprises 22 items grouped into four dimensions: proficiency, altruism, prevention, and leadership. The authors suggest that the NCSES, when assessing nursing students’ self-efficacy, can be a valid tool for determining the competencies that graduates acquire in their training and their innovative potential toward the profession. Also focused on nursing students’ self-efficacy, Oetker-Black et al. [13] developed the Clinical Skills Self-Efficacy Scale (CSES), and Zengin et al. [14] validated the Self-Efficacy for Clinical Evaluation Scale (SECS) in a Turkish nursing student sample.

These works to develop skills assessment tools have been extended to specific areas of clinical practice. Two examples are the nurses’ core competence in palliative care (NCPC; [15]) or the questionnaire to assess nursing competencies for the care of people with psychiatric disabilities in a hospital environment [16]. The evaluation of specific competencies has also been an objective of the research, with works such as the development of the Compassion Competence Scale [17] or the Nurse Cultural Competence Scale (NCCS; [18]) or the EPICC Spiritual Care Competency Self-Assessment Tool [19].

The abovementioned studies show an interest in developing practical tools for evaluating competencies inherent to the nursing field. In the context of the EHEA, universities have made a significant effort to establish objective and effective assessment systems for the competencies developed by students in professional settings.

Although self-assessment is considered an essential part of the evaluation of the clinical practice of nursing students [7,20], very few systems consider competency self-assessments by nursing students throughout their career, and those that are developed do not usually have an impact on the final assessment at the end of the clinical practice. However, Andrade proposes that self-assessment can be summative; the evidence suggests that it is most beneficial “in terms of both achievement and self-regulated learning, when it is applied formatively and supported by training” [21] (p. 10).

Based on these premises, the need to develop a reliable and valid measurement instrument for self-evaluation by students of their self-efficacy expectations in nursing competencies at the end of their clinical placement of clinical practicum is addressed. This general objective can be broken down into the following specific objectives: (a) to analyze the psychometric properties of the PSENC Scale; (b) to explore and conceptualize the possible dimensions that make up the construct of Self-Efficacy Expectations in Nursing Competencies; (c) to examine whether there are significant differences in the judgment made by students of different courses about their level of competence development during the clinical practicum.

## 2. Materials and Methods

For the development of the PSENC Scale, the 40 specific competencies proposed by the Tuning Project [5] for nursing degrees, which have been assumed in the study plans of this degree in the EHEA, were used. For the selection of the items, the wording of each of the competencies was analyzed and proceeded to (1) unfold those competencies that measured two or more related but differentiated aspects in their content and (2) adapt the wording to facilitate the understanding of the conceptual meanings of competencies by students. As a result, a relation of 51 items was presented for evaluation to two groups of students from the second and third academic years of a four-year program. Students were asked to express their sense of the conceptual content of each competency and the difficulties they perceived in understanding them. With their contributions, the wording of the proposed sentences was revised and modified to increase their clarity and understanding.

From now on, and to evaluate content validity, the set of competencies was presented to a group of experts (eight university professors and nine nursing professionals related to practical training). These experts checked the wording for interpretation problems and suggested alternative wording to improve their understanding and uniqueness. They were also asked to judge whether the content of each sentence was relevant to the purpose of the scale.

The resulting 51-item prototype evaluated the indicators using an 11-point Likert-type scale, where 0 would be equivalent to “incapable” and 10 to “totally capable”. For European samples, “the use of items measured by an 11-point scale leads to composite scores with higher reliability and lower invalidity than the use of a 5-point scale” [22] (p. 580). Students then submitted the online version of the instrument. From the analysis of the data and the results obtained, we proceeded to study the reliability and validity of the scale.

### 2.1. Participants and Data Collection

The online PSENC Scale was submitted to 1811 nursing degree students from six Spanish universities between September 2013 and October 2022 and was answered entirely by 1416 students. The instrument was developed within a project to study the determinants of student well-being in the clinical nursing practicum. The project was presented to the students, and the only exclusion criterion was that the student needed to be enrolled in the subject corresponding to the clinical practicum of their academic year. Participation was voluntary, and they answered the instrument at the end of their clinical placement. They also gave their informed consent for their data to be incorporated into a file for the project’s development.

### 2.2. Ethical Considerations

The study was assessed and approved by the Ethical Committee of the Comillas Pontifical University. The procedures to obtain, process, and communicate the data from this research were aligned with the provisions outlined in the European regulation and Spanish legislation on personal data protection.

### 2.3. Data Analysis

The measurement instrument was validated in the first stage by reliability and exploratory factor analysis (EFA) techniques. The total sample was randomly distributed into two groups: one for EFA and another for confirmatory factor analysis (CFA). For the EFA, an estimate was made from the polychoric correlation matrix using the Unweighted Least Squares (ULS) method, the most appropriate option given the circumstance of the polytomous nature of the items and the lack of normality of the distribution.

Based on the dimensions resulting from the EFA and in congruence with the theoretical framework of the research, two models were proposed for its CFA using structural equation modeling (SEM) techniques. To check the goodness of fit and the validity of the models, the normed χ2 statistic and contrasted descriptive indexes of the degree of fit were used. The computer program used for the EFA from polychoric correlations was the Factor 10.8.04 program [23]. The EQS 6.2 software for Windows was used for the model CFA. The different fit indices and residuals have been calculated by the robust maximum likelihood estimation method, which is less sensitive to the absence of multivariate normality (Mardia’s coefficient > 5) presented by the distributions of the data obtained.

For the inferential analysis, the subsamples generated from the categories gender, employment situation, age range, and academic level were considered. The analysis used the non-parametric Kruskal–Wallis test, contrasted with the multivariate analysis of variance techniques (Bonferroni post hoc test), using IBM SPSS Statistics 28.0.1.1 program.

## 3. Results

The 1416 students who completed the online questionnaire represent a valid response rate of 75.19%. A total of 607 (42.9%) reported being in the second year of the nursing degree, 613 (43.3%) in the third year, and 196 (13.8%) in the fourth year. The average age of the participants was 24.15 ± 5.22 years old. Regarding gender, 1203 were women (85%) and 213 men (15%). Finally, 329 students have combined a work activity during the academic year (23.2%). The general characteristics of the participants are shown in Table 1.

### 3.1. Reliability and Exploratory Factor Analysis (EFA)

The EFA carried out on the final prototype of the 51-item scale made it possible to identify that the most significant indicators were grouped into five dimensions. The result was a 20-item scale (Tables S1 and S2) after reviewing the conceptual contents of the items based on the theoretical foundation and selecting those that maintained the most significant weights in each of the factors without sharing appreciable weights in the rest.

This scale presented a high internal consistency index from the complete sample data (Cronbach’s α value = 0.927, and the mean inter-item correlations = 0.41).

The EFA was conducted on a random split-half sample of the data (N = 712) to examine the factor structure of the 20 PSENC Scale items. When the EFA was applied to the scale, the Kaiser–Meyer–Olkin (KMO) “sampling adequacy” index showed a value of 0.934 (close to 1), and Bartlett’s Test of Sphericity was significant (χ2 = 8911.0; df = 190; *p* < 0.00001).

In the solution obtained in the EFA (Table S3) from the matrix of polychoric correlations on the responses to the scale (ULS and rotation Oblimin direct), five factors were identified in the extraction that explains 76.9% of the total variance, with adequate fit indices: Comparative Fit Index (CFI) = 1.001; Tucker and Lewis Index (TLI) = 1.003. The decision to retain five factors in the extraction was corroborated by the values presented by the Root Mean Square Error of Approximation (RMSEA) = 0.000 and the Goodness of Fit Index (GFI) = 0.999, which are acceptable values for the goodness of fit indices, and the value of the Root Mean Square of Residuals (RMSR) = 0.017, which was lower than the expected mean value of RMSR (0.037) according to Kelley’s criterion [24]. The five subscales presented high internal consistency indexes with Cronbach’s α values between 0.774 and 0.910, which are adequate considering the low number of items in each factor. The homogeneity indices were also satisfactory, with item-total correlations higher than 0.47 in each indicator. Therefore, the proposed items aim to identify between-subject differences in the factors resulting from this exploration.

From these results, the resulting latent variables were conceptualized based on the observable variables. This situation affirmed that the construct Expectations of Self-Efficacy in Nursing Competencies could be structurally configured into five components or dimensions: (a) Knowledge (about interrelated data that allow the application of various fields of knowledge in nursing practice); (b) Critical Thinking (logical and analytical thinking skills designed to make clinical judgments); (c) Intervention (ability to carry out direct care aimed at the person, the family, and the community that favors the expected results outlined in the users’ care plans); (d) Ethics of Care (ethical skills related to the cognitive processes that generate the positioning of the nursing professional in the face of ethical or moral dilemmas that may arise in their practice and the qualities it confers on the process of caring for people); and (e) Communication (communication skills and their development, encompassing relationships with users and with the health team). All the resulting factors maintained important intercorrelations. A second-order EFA showed that the factors had a one-dimensional factorial structure (Table 2).

Consequently, a factor of second order was obtained as a factorial synthesis of the 20 indicators, which explained 53.96% of the variance and was conceptually interpreted as “Expectations of self-efficacy in nursing competencies”. The construct can be defined operationally as the “judgments that the individual makes about their capacities to reach a certain level of performance that allows them to confront problem situations safely in the context of the clinical nursing practicum based on the dynamic combination of attributes with respect to knowledge and its application, to attitudes and responsibilities”.

### 3.2. Confirmatory Factor Analysis (CFA)

A CFA was conducted in the second random sample (N = 704) to confirm the factor structure. Two rival measurement models were evaluated on the sample, which was plausible from the theoretical and empirical points of view. The results suggest that the model with five correlated factors presents more satisfactory fit indices (Table 3). The Satorra–Bentler scaled χ2 statistic was significant, which is common in a large sample (S-B χ2 = 505.32; d.f. = 160; *p* < 0.001). The parsimony adjustment of the model (normed chi-square = 3.16) was within the recommended levels. The Normed Fit Index (NFI) showed a value = 0.890, the TLI or Non-Normed Fit Index (NNFI) = 0.907, and the CFI = 0.922, all the above indicating satisfactory goodness of fit indices. However, the RMSEA was 0.055, with a 90% confidence interval of 0.050 to 0.061, which indicated a good fit.

**Figure 1:**
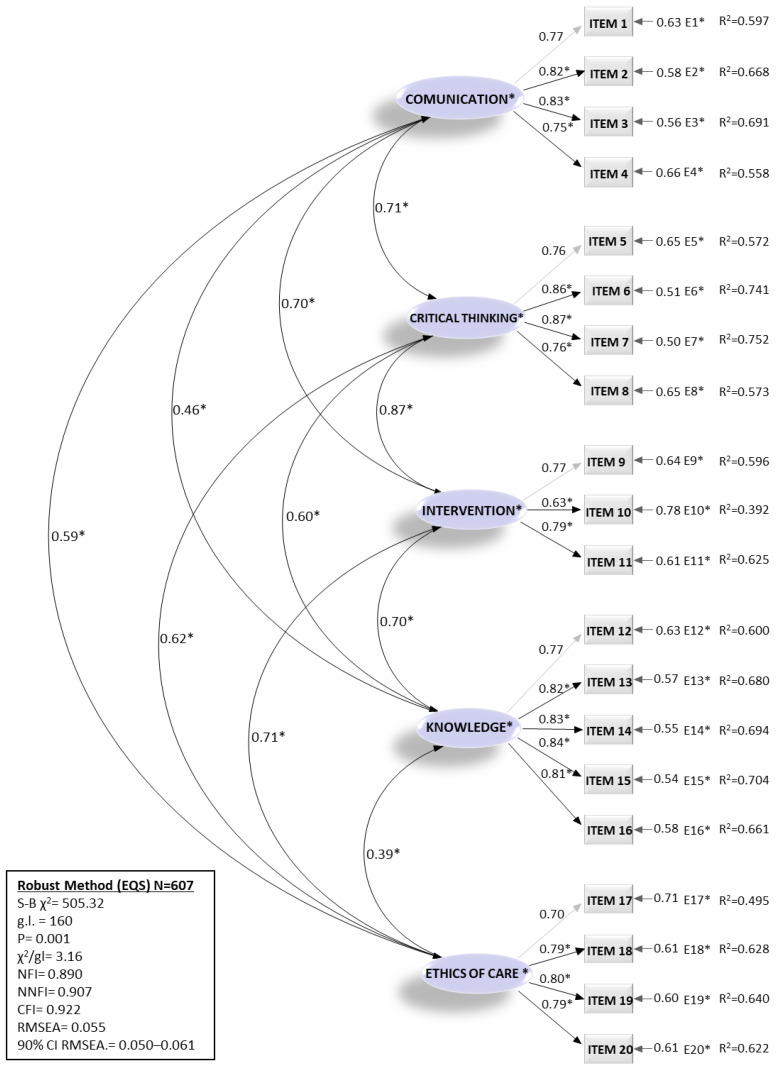
Standardized parameter estimates for the measurement model of the PSENC Scale. Model 2 (five correlated factors). (N = 704); * *p* < 0.001.

It can be concluded that all the calculated goodness of fit indices looks suitable between the postulated theoretical model and the sample data. In a more in-depth analysis of the proposed model’s standardized solution (Figure 1), we established that all parameters measured were positive and significant.

The indicators show adequate reliability, with factor loadings greater than 0.62 and R^2^ greater than 0.49, and a Composite Reliability (CR) in each construct with values between 0.67 and 0.91. The Average Variance Extracted (AVE) of the factors’ presented values between 0.40 and 0.66, which together with values > 0.70 in the CR, indicate adequate convergent validity [25]. Finally, the following was confirmed: the discriminant validity of the construct checking that the square root of AVE was increased compared to the correlations with other latent constructs [26] and that the Maximum Shared Variance (MSV) and Average Shared Variance (ASV) values were less than the AVE, according to the standards recommended by Hair et al. [25].

### 3.3. Inferential Analysis

The Kruskal–Wallis test was used, contrasted with multivariate analysis of variance techniques, to determine the differences in the perception of self-efficacy in nursing competencies of the nursing degree students according to their academic year.

In the results shown in Table 4 (the academic years with which they present significant differences are shown in parentheses; Bonferroni post hoc test, *p* < 0.05), it can be verified that there are significant differences between the average score levels of the students regarding the perception of self-efficacy in all groups of competencies according to their academic year. It should be noted that although the average scores obtained are linked to the level of specific competence development for each academic year, the final-year students present the best scores in the five dimensions.

Regarding perceived self-efficacy in nursing competencies related to age ranges, the significant differences between the subsamples are shown in Table 5.

Concerning the employment situation, students who combine their study with a work activity present a significantly higher average perception in communication, critical reasoning, and knowledge competencies and are significantly lower in ethical care competencies. In the gender analysis, females showed a significantly higher average perception of the ethics of care competencies. All cases presented small effect sizes.

## 4. Discussion

This article evaluates the construct validity and internal consistency of the PSENC Scale for the self-evaluation of self-efficacy expectations in nursing competencies by students at the end of their training stays at the clinical practicum.

Composed of 20 items, the PSENC Scale displays a high internal consistency (Cronbach’s alpha = 0.932, and the five subscales’ coefficients between 0.73 and 0.93). The results obtained by the EFA and CFA techniques confirm the factorial structure of the scale and the construct validity. The CFA results allow us to conclude that the construct self-efficacy expectations in nursing competencies in the clinical practicum are made up of five factors that explain 76.3% of the total variance, which we have conceptualized as “Communication”, “Critical Thinking”, “Intervention”, “Knowledge”, and “Ethics of Care”. Therefore, the evaluation of the psychometric characteristics of the scale has confirmed the factorial structure through the proposed measurement model, which presented a good fit on all indices.

In addition, these factors coincide in four cases with the grouping of competencies proposed by the Tuning Project Group for Nursing [5]: nursing practice and clinical decision-making (Critical Thinking and Intervention), knowledge and cognitive competencies (Knowledge), communication and interpersonal competencies (Communication). The competencies corresponding to the items that make up the “Nursing ethics” factor are distributed among the different groups proposed by the Tuning Project. They are also in line with the philosophy of competence in nursing proposed by Franklin and Melville [27], where knowledge, skills, attitudes, and clinical judgment are interrelated in implementing safe, evidence-based patient interventions.

The competence self-assessment of nursing students throughout their careers is relevant. It should be noted that the relationship between capacity self-assessments framed in a specific domain maintains a more robust relationship with objective performance assessments (e.g., evaluations by preceptor) than those in more global contexts [28]. In this sense, the study by Adib-Hajbaghery et al. [29] showed that the mean scores of the self-assessments of nursing students in clinical skills significantly correlated with the scores they received from educators. Instruments for competence self-assessment throughout their career provide important information to educators in their orientation towards a progressive acquisition of professional skills. The PSENC Scale can provide relevant information in this regard. It also guides students and preceptors on the levels of the acquisition of competence that, in each case, require special attention, favoring the improvement of learning dynamics in the clinical nursing practicum.

At the same time, and within the framework of the Social Cognitive Theory, students’ self-evaluation of their self-efficacy expectations in competencies can provide critical information to teachers. Moreover, it is vital for the role that they develop before three essential sources of self-efficacy beliefs [30,31]: mastery experiences (evaluation of the commitment to achieve success), social persuasion (evaluative feedback), and physical and emotional states (promoting the perception that these reactions are the result of effort). Bandura does not attribute these sources a determining value by themselves [32]. Its importance lies in the subjects’ cognitive processing, since the information they provide is selected, evaluated, and integrated into their self-efficacy judgements.

Regarding the inferential analysis, the average perception of a level of competence between very good and excellent of fourth-year students can be considered predictable, coinciding with research on graduating nursing students by Forsman et al. [33], Kajander-Unkuri et al. [34], Visiers-Jiménez et al. [35], and Wangensteen et al. [36]. Here, the high level of perception of competence on the part of the students, especially those in the second year, is noteworthy. These results are similar to those of the study by Nemcová et al. [37], in which the self-assessments of the students’ levels of competence showed an average perception of good levels of competence in the overall results.

### Limitations

The sample’s incidental nature limits the instrument’s external validity, degrading its ability to generalize the results and conclusions. Having a representative sample of other European universities in future research would allow us to have a solid observatory of the clinical nursing practicum and its specificities in the framework that represents the EHEA. The representativeness of fourth-year students could be considered low. Finally, in the field of competency assessments, it would also be necessary to consider the assessment of educators in future research.

## 5. Conclusions

In this study, a new nursing competency self-efficacy scale was developed. The results shown by the PSENC Scale allow us to affirm that the instrument can facilitate the self-assessment of the nursing competence self-efficacy of students with sufficient reliability and validity. With only 20 items and a structure of five factors that explain 76.3% of the variance, it is configured as a simple instrument to administer. Additionally, it can provide educators and researchers with crucial information that allows them to guide and evaluate curricular initiatives to develop nursing competency self-efficacy in students.

## Figures and Tables

**Table 1:** Socio-demographic characteristics of participants (N = 1416).

Gender	Age Range (Years)				
	19–24	25–30	31–40	>40	Total
Female	858 (60.6%)	235 (16.6%)	89 (6.3%)	21 (1.5%)	1203 (85%)
Male	119 (8.4%)	66 (4.7%)	17 (1.2%)	11 (0.7%)	213 (15%)
Employed	174 (12.3%)	86 (6.1%)	48 (3.4%)	21 (1.5%)	329 (23.3%)
Total	977 (69%)	301 (21.3%)	106 (7.5%)	32 (2.2%)	1416

**Table 2:** Second-order factor structure of the Self-Efficacy Expectations in Nursing Competencies construct. PSENC scale factors and correlation matrix. (N = 712).

Self-Efficacy Expectations in Nursing Competencies	Component	1	2	3	4
1. Intervention	0.836	1			
2. Critical Thinking	0.834	0.683 *			
3. Communication	0.715	0.553 *	0.619 *		
4. Knowledge	0.622	0.571 *	0.552 *	0.397 *	
5. Ethics of Care	0.638	0.552 *	0.493 *	0.529 *	0.339 *

Extraction Method: Unweighted Least Squares; Rotation Method: Direct Oblimin. * Correlation is significant at the 0.01 level (2-tailed).

**Table 3:** Goodness of fit indicators for the hypothesized models (N = 704).

Model	Satorra–Bentler χ2 ^1^	df	NFI Robust	NNFI Robust	CFI Robust	RMSEA Robust
Model 1: Four correlated factors	595.51	164	0.870	0.887	0.902	0.061 (0.056, 0.066)
Model 2: Five correlated factors	505.32	160	0.890	0.907	0.922	0.055 (0.050, 0.061)

^1^ *p* < 0.001.

**Table 4:** Perceived self-efficacy in nursing competencies related to the academic year. Multivariate Kruskal–Wallis test with post hoc (N = 1416).

Factors	Academic Year	N	$\bar{X}$	σ	df	Kruskal–Wallis Test		Ɛ^2^R
						H	*p*	
Communication	1. Second	607	8.38	1.06	2 1413 1416	8.57	0.014	0.006
	2. Third	613	8.31 _(3)_	1.06				
	3. Fourth	196	8.56 _(2)_	1.02				
	Total	1416	8.37	1.06				
Critical Thinking	1. Second	607	7.79 _(2) (3)_	1.13	2 1413 1416	35.64	0.001	0.023
	2. Third	613	7.95 _(1) (3)_	1.02				
	3. Fourth	196	8.29 _(1) (2)_	0.87				
	Total	1416	7.93	1.06				
Intervention	1. Second	607	8.11 _(3)_	1.16	2 1413 1416	20.20	0.001	0.014
	2. Third	613	8.24 _(3)_	1.07				
	3. Fourth	196	8.50 _(1) (2)_	0.99				
	Total	1416	8.22	1.10				
Knowledge	1. Second	607	6.56 _(2) (3)_	1.94	2 1413 1416	62.36	0.001	0.049
	2. Third	613	7.09 _(1) (3)_	1.52				
	3. Fourth	196	7.68 _(1) (2)_	1.29				
	Total	1416	6.94	1.73				
Ethics of Care	1. Second	607	8.80 _(3)_	0.97	2 1413 1416	22.47	0.001	0.014
	2. Third	613	8.87 _(3)_	1.05				
	3. Fourth	196	9.16 _(1) (2)_	0.84				
	Total	1416	8.88	1.00				

The academic years with which they present significant differences are shown in parentheses (Bonferroni post hoc test; *p* < 0.05). The number in parentheses refers to the corresponding academic year: 1. Second: _(1)_; 2. Third: _(2)_; 3. Fourth: _(3)_.

**Table 5:** Perceived self-efficacy in nursing competencies related to age. Multivariate Kruskal–Wallis test with post hoc (N = 1416).

Factors	Age Ranges	N	$\bar{X}$	σ	df	Kruskal–Wallis Test		Ɛ^2^R
						H	*p*	
Communication	1. 19–24	977	8.33 _(3) (4)_	1.04	3 1413 1416	14.55	0.002	0.010
	2. 25–30	301	8.41 _(4)_	1.09				
	3. 31–40	106	8.58 _(1)_	1.04				
	4. >40	32	8.71 _(1) (2)_	1.35				
	Total	1416	8.37	1.06				
Critical Thinking	1. 19–24	977	7.89 _(2)_	1.02	3 1413 1416	7.00	0.072	0.005
	2. 25–30	301	8.03 _(1)_	1.07				
	3. 31–40	106	8.01	1.32				
	4. >40	32	7.91	1.40				
	Total	1416	7.93	1.06				
Intervention	1. 19–24	977	8.18 _(2)_	1.08	3 1413 1416	5.72	0.126	0.005
	2. 25–30	301	8.31 _(1)_	1.09				
	3. 31–40	106	8.31	1.23				
	4. >40	32	8.23	1.36				
	Total	1416	8.22	1.10				
Knowledge	1. 19–24	977	6.89	1.73	3 1413 1416	3.65	0.302	0.002
	2. 25–30	301	7.05	1.65				
	3. 31–40	106	6.98	1.83				
	4. >40	32	7.32	1.84				
	Total	1416	6.94	1.73				
Ethics of Care	1. 19–24	977	8.89	0.95	3 1413 1416	1.90	0.594	0.001
	2. 25–30	301	8.84	1.05				
	3. 31–40	106	8.90	1.21				
	4. >40	32	8.98	1.17				
	Total	1416	8.88	1.00				

The academic years with which they present significant differences are shown in parentheses (Bonferroni post hoc test; *p* < 0.05). The number in parentheses refers to the corresponding age ranges: 1. 19–24: _(1)_; 2. 25–30: _(2)_; 3. 31–40: _(3)_; 4. >40: _(4)_.

## Data Availability

The original contributions presented in the study are included in the article/Supplementary Material; further inquiries can be directed to the corresponding author/s.

## Supplementary Materials

The following supporting information can be downloaded at https://www.mdpi.com/article/10.3390/nursrep14030173/s1. Table S1: Perceived Self-Efficacy in Nursing Competencies (PSENC) Scale (English version); Table S2: Perceived Self-Efficacy in Nursing Competencies (PSENC) Scale (Spanish version); Table S3: Factor loading of each item of the PSENC Scale. Rotated loading matrix. Variance explained and Cronbach’s α (N = 712).

## Public Involvement Statement

No public involvement in any aspect of this research.

## Guidelines and Standards Statement

This manuscript was drafted against the STROBE (recommendations for reporting the results of studies of instrument and scale development and testing [38]) for observational studies research.
