# Supplementary material for: Self-Efficacy in Nursing Competencies during Students’ Clinical Practicum: The Development of a Self-Assessment Scale
Source: Nurs Rep. 2024 Sep 10;14(3):2327–39. doi: 10.3390/nursrep14030173 (PMC11417760; doi:10.3390/nursrep14030173)
Supplement: Supplementary file 1 [file nursrep-14-00173-s001.zip › nursrep-3104830-supplementary.pdf]

**Table S1.** Perceived Self-Efficacy in Nursing Competencies (PSENC) Scale (English version)

We present below a series of competencies to reflect the level of development that has been achieved in each of the participants during this course of the degree.
Rate on a scale of 0 to 10 until the LEVEL YOU THINK YOU ARE CAPABLE OF:

1. Communicate effectively with patients.
2. Make it easy for patients to express their feelings and concerns.
3. Properly use communication skills to counsel the patients.
4. Provide emotional support to the patient.
5. Undertake systematic health assessments covering all dimensions of the person (physical–social–cultural–psychological–spiritual).
6. Recognize clinical signs indicating a person’s level of health/well-being.
7. Interpret clinical signs indicating a person’s level of health/well-being.
8. Make my clinical judgments based on quality information and with sufficient scientific rigor.
9. Apply the skills to implement (put into practice) health and safety principles (mobilization, infections, first aid, and emergency procedures).
10. Safely administer medicines and other therapies.
11. Meet the specific needs of people who experience an illness and its consequences adapting to their life stage.
12. Mastering the necessary knowledge to apply principles of legislation to professional practice.
13. Mastering the necessary knowledge to apply technological innovations to health care.
14. Mastering the necessary knowledge to apply the principles of national and international politics to professional practice.
15. Mastering the skills needed to generate problems solving and decision-making strategies.
16. Mastering the necessary knowledge to apply scientific principles backed by accredited sources of information.
17. Accept that the patient’s well-being is achieved through the coordinated work of the social–health care team.
18. Approach care holistically, tolerantly, without prosecution, carefully, and sensitively.
19. Maintain patient dignity, privacy, and confidentiality.
20. Adequately defend the patient’s perspective and act, if necessary, to prevent abuse.

**Table S2.** ESCALA DE AUTOEFICACIA PERCIBIDA EN COMPETENCIAS DE ENFERMERÍA (Perceived Self-Efficacy in Nursing Competencies Scale [PSENC]) (Spanish version)

Te presentamos a continuación una serie de competencias para que reflexiones sobre el nivel de desarrollo que has conseguido en cada una de ellas durante este curso de la titulación

Valora en la escala de 0 a 10 hasta que NIVEL TE CREES CAPAZ DE:

1. Comunicarte de manera efectiva con los usuarios.
2. Facilitar que los usuarios expresen sus sentimientos y preocupaciones.
3. Utilizar de forma adecuada las habilidades de comunicación para asesorar al usuario.
4. Dar apoyo emocional al usuario.
5. Realizar valoraciones de salud que cubran todas las dimensiones de la persona (biológica-psicológica-social-cultural-espiritual) de forma sistemática.
6. Identificar los signos clínicos que indican el grado de salud/bienestar de la persona.
7. Interpretar los signos que indican el grado de salud/bienestar de la persona.
8. Elaborar mis juicios clínicos apoyándome en una información de calidad y con el suficiente rigor científico.
9. Aplicar las habilidades para poner en práctica principios de salud y seguridad (movilización, infecciones, primeros auxilios y procedimientos de emergencia).
10. Administrar con seguridad fármacos y otras terapias.
11. Responder a las necesidades específicas de personas que experimentan una enfermedad y sus consecuencias adaptándome a su etapa vital.
12. Dominar los conocimientos necesarios para aplicar principios de legislación a la práctica profesional.
13. Dominar los conocimientos necesarios para aplicar las innovaciones tecnológicas a los cuidados de salud.
14. Dominar los conocimientos necesarios para aplicar los principios de la política nacional e internacional a la práctica profesional.
15. Dominar los conocimientos necesarios para generar estrategias de resolución de problemas y toma de decisiones.
16. Dominar los conocimientos necesarios para aplicar principios científicos apoyados en fuentes de información acreditadas.
17. Aceptar que el bienestar del paciente se alcanza a través del trabajo coordinado del equipo sociosanitario de cuidados.
18. Abordar el cuidado de manera holística, tolerante, sin enjuiciamientos, cuidadosa y sensible.
19. Mantener la dignidad, privacidad y confidencialidad del paciente.
20. Defender adecuadamente la perspectiva del paciente y actuar, si es necesario, para evitar abusos.

**Table S3.** Factor loading of each item of the PSENC Scale. Rotated loading matrix. Variance explained and Cronbach's α (N=712).

| | | | $\bar{X}$ | σ | F1 | F2 | F3 | F4 | F5 | Communality | Variance Explained |
|---|---|---|---|---|---|---|---|---|---|---|---|
| | | | | | Components | | | | | | |
| Ethics of Care | α = 0.832 | Item 18 | 8.73 | 1.31 | 0.802 | 0.011 | 0.002 | 0.040 | -0.009 | 0.668 | 3.55% |
| | | Item 19 | 9.22 | 1.08 | 0.876 | 0.020 | 0.045 | -0.066 | -0.049 | 0.752 | |
| | | Item 20 | 8.75 | 1.29 | 0.672 | 0.077 | -0.025 | 0.154 | 0.070 | 0.654 | |
| | | Item 17 | 8.83 | 1.20 | 0.592 | 0.022 | 0.021 | -0.014 | 0.178 | 0.514 | |
| Communication | α = 0.865 | Item 2 | 8.32 | 1.27 | -0.116 | 0.892 | 0.028 | -0.010 | 0.034 | 0.751 | 11.66% |
| | | Item 1 | 8.57 | 1.20 | 0.122 | 0.706 | -0.130 | 0.001 | 0.090 | 0.606 | |
| | | Item 3 | 8.14 | 1.24 | 0.096 | 0.760 | 0.103 | 0.052 | -0.064 | 0.739 | |
| | | Item 4 | 8.47 | 1.31 | 0.053 | 0.701 | -0.011 | 0.052 | 0.063 | 0.633 | |
| Critical Thinking | α = 0.875 | Item 6 | 8.06 | 1.17 | -0.000 | 0.031 | 0.865 | -0.039 | 0.053 | 0.805 | 5.29% |
| | | Item 7 | 8.11 | 1.18 | 0.066 | -0.029 | 0.952 | 0.005 | -0.062 | 0.865 | |
| | | Item 5 | 7.85 | 1.31 | -0.126 | 0.204 | 0.557 | 0.081 | 0.084 | 0.553 | |
| | | Item 8 | 7.69 | 1.32 | -0.040 | 0.059 | 0.514 | 0.256 | 0.174 | 0.684 | |
| Knowledge | α = 0.910 | Item 14 | 6.23 | 2.38 | -0.057 | -0.002 | 0.009 | 0.991 | -0.069 | 0.897 | 7.21% |
| | | Item 12 | 6.52 | 2.29 | 0.011 | 0.048 | -0.025 | 0.910 | -0.050 | 0.800 | |
| | | Item 13 | 7.21 | 1.89 | 0.094 | -0.043 | 0.038 | 0.753 | 0.124 | 0.746 | |
| | | Item 16 | 7.19 | 1.82 | 0.064 | 0.023 | 0.051 | 0.710 | 0.126 | 0.723 | |
| | | Item 15 | 7.57 | 1.56 | 0.086 | 0.038 | 0.107 | 0.564 | 0.234 | 0.717 | |
| Intervention | α = 0.774 | Item 9 | 8.24 | 1.24 | 0.050 | 0.088 | 0.163 | 0.006 | 0.625 | 0.669 | 49.21% |
| | | Item 10 | 8.25 | 1.42 | 0.181 | -0.019 | 0.056 | 0.137 | 0.447 | 0.446 | |
| | | Item 11 | 8.17 | 1.32 | 0.109 | 0.128 | 0.045 | 0.147 | 0.575 | 0.693 | |
| **Cronbach's =0.927** | | | | | | **Total Variance Explained** | | | | | **76.92%** |

Extraction method: Unweighted Least Squares. Dispersion matrix: Polychoric Correlations.
Rotation method: Oblimin direct.
Factor loading coefficients below 0.3 were suppressed.
